# Revisiting the prevalence of bipolar disorder: implications of a broader spectrum model

**DOI:** 10.3389/fpsyt.2026.1776491

**Published:** 2026-03-23

**Authors:** Anatoly Kreinin

**Affiliations:** Clalit Psychiatric Hospitalization Control System, Clalit General Health Services, Israel Adelson School of Medicine, Ariel University, Ariel, Israel

**Keywords:** ADHD, bipolar affective disorders, bipolar spectrum disorders, borderline personality disorder, comorbidities, substance use disorders

## Abstract

Bipolar affective disorder has traditionally been defined by episodic mania and depression, largely restricted to Bipolar I and II categories. However, growing evidence suggests this framework underrepresents the broader longitudinal spectrum and may misclassify a subset of cases as borderline personality disorder (BPD) or other comorbidities. A narrative review was conducted, integrating historical and conceptual analyses of diagnostic frameworks (DSM-III to DSM-5), epidemiological studies over four decades, and the author’s extensive clinical experience with longitudinal follow-up of complex affective cases. Literature addressing comorbidity, diagnostic delays, and symptom overlap was critically examined within a comparative and hypothesis-oriented framework. Findings indicate that bipolar spectrum disorders (BPSD) are substantially underdiagnosed, with prevalence plausibly exceeding traditional categorical estimates when cumulative longitudinal spectrum phenomena are considered. The suggested prevalence range of 15–20% represents a theoretical upper-bound hypothesis derived from clinical extrapolation and longitudinal observation, rather than a definitive epidemiological estimate. Conditions commonly diagnosed as BPD, ADHD, or anxiety may, in a subset of cases, represent longitudinal affective presentations overlapping with the bipolar spectrum. Evidence supports a spectrum model encompassing multiple subtypes, such as bipolar with panic attacks, ADHD comorbidity, antidepressant-induced mania, and ultra-rapid cycling. The continued use of BPD as a diagnostic category is questioned due to poor construct validity and overlap with bipolarity. Reconceptualizing bipolar disorder as a spectrum within a cautiously framed longitudinal model is essential to improve diagnostic accuracy, guide treatment, and optimize long-term outcomes, while remaining subject to prospective empirical validation. This manuscript is explicitly framed as a narrative, hypothesis-generating review rather than a systematic review or meta-analysis; accordingly, prevalence estimates discussed herein should be regarded as exploratory and conceptual rather than definitive epidemiological claims.

## 1 Methodological framework

This manuscript is designed as a narrative, hypothesis-generating review rather than a systematic review or meta-analysis. Literature was identified through targeted searches of PubMed and PsycINFO using thematic keywords including bipolar spectrum, subthreshold bipolarity, cyclothymia, affective instability, and diagnostic instability. Priority was given to landmark longitudinal studies, authoritative psychiatric textbooks, and influential conceptual models in affective psychopathology.

The aim of this article is not to redefine diagnostic criteria, but to examine whether current nosological frameworks systematically underestimate bipolar spectrum phenomena, thereby limiting diagnostic sensitivity and clinical understanding.

To minimize selection bias, contrasting perspectives were intentionally included, including DSM-based categorical models that do not support spectrum expansion. The goal was conceptual integration and critical analysis rather than exhaustive literature coverage.

Throughout this article, prevalence estimates referring to bipolar spectrum conditions are presented as indirect and hypothesis-generating rather than definitive epidemiological figures. Variability in diagnostic definitions, study design, sampling methods, and longitudinal follow-up contributes to substantial heterogeneity across reported rates. Accordingly, numerical estimates are interpreted in a comparative and conceptual framework rather than as fixed population prevalences. This variability reflects not only methodological differences across studies, but also the inherent instability of nosological boundaries in affective psychiatry, where longitudinal course frequently reshapes cross-sectional diagnostic assignments.

Much of the conceptual foundation of bipolar spectrum psychopathology derives from classical longitudinal and phenomenological studies conducted more than two decades ago. While these earlier works remain influential in shaping current nosological debates, their findings must be interpreted within their historical and methodological context. Accordingly, in the present analysis, such studies are cited primarily for their conceptual and heuristic value rather than as sources of definitive epidemiological estimates. Where available, their conclusions are balanced with more recent large-scale epidemiological investigations, systematic reviews, and contemporary cohort studies employing updated diagnostic frameworks. This integrative approach aims to preserve the theoretical insights of foundational literature while avoiding overgeneralization and ensuring consistency with current empirical evidence. Contemporary reviews have emphasized that the apparent low prevalence of bipolar disorder is largely a function of restrictive diagnostic thresholds rather than true rarity of the condition (1).

In this context, the conceptual decomposition model presented later in this manuscript should be understood not as a statistical extrapolation per se, but as a heuristic effort to model how longitudinal affective phenomena-when systematically incorporated into epidemiological reasoning-may alter lifetime prevalence estimates. The proposed upper-bound scenario therefore reflects a theoretical modeling exercise grounded in longitudinal phenomenology rather than a fixed epidemiological assertion.

## 2 Introduction: historical background

One of the earliest references to bipolar affective disorder, music therapy and self-medication for depression is in the Bible. King Saul experienced mood fluctuations and unstable behavior which were eased when David played his harp, A depiction of mania, and even the term, appear in Homer’s Iliad in the description of Achilles’ uncontrollable rage toward Agamemnon (2, 3).

Bipolar affective disorder is characterized by alternating periods of mania and depression. In the 19th century, Jean-Pierre Falret coined the term “circular insanity” to describe the cyclic nature of the illness. Jules Baillarger described the disorder characterized by alternating manic and depressive states, calling it *la folie à double forme* (4). In 1899 Kraepelin distinguished this illness from other psychotic states, naming it Manic-Depressive Psychosis (MDP) (5). In the 1950s, Karl Leonhard introduced the concept of polarity in affective disorders (6).

Modern classification – along with the term “bipolar affective disorder” – was established in the late 1970s. Conceptualization of the illness is tied to the development of valid diagnostic criteria during the 1970s (7). Following the introduction of the DSM-III in 1980 by the American Psychiatric Association, the medical community accepted the DSM criteria for Bipolar I Disorder.

In the 1960s, Jules Angst, a Swiss psychiatrist, differentiated between subtypes of bipolar disorders and emphasized the differences between Bipolar I and Bipolar II, with the latter characterized by hypomanic episodes and major depressive episodes (7, 8).

Over time, a broader category known as Bipolar Spectrum Disorders (BSD) emerged (9) (**Table 1**).

**Table 1:** Historical bipolar spectrum prototypes (adapted from Akiskal and Pinto, 10).

• Bipolar I - Mania + Depression • Bipolar II - Hypomania + Depression • Bipolar III - Antidepressant-associated hypomania • Bipolar IV - Hyperthymic temperament + depression • Bipolar II½ - Cyclothymic temperament + major depression • Minor bipolar disorders (subthreshold hypomania)

Historical conceptual models; not formal DSM classification.

## 3 Epidemiology

Pope and Lipinski (11) conducted the first notable epidemiological study focusing on bipolar disorder in 1978 and examined the frequency of diagnosing schizophrenia versus bipolar disorder. They found a diagnostic ratio of 8:1 in favor of schizophrenia in the U.S., compared to a 1:1 ratio in Western Europe (11).

In 1977, Woodruff et al. (12) proposed strategies to reduce diagnostic ambiguity between Bipolar I Disorder and schizophrenia. They recommended prioritizing affective disorder diagnoses due to their association with more favorable treatment outcomes – reflecting growing recognition of bipolar disorder as a distinct clinical entity.

Epidemiological studies based on narrowly defined DSM criteria report prevalence rates of approximately 1–4%. However, population-based surveys employing broader spectrum definitions have identified substantially higher rates of bipolar spectrum conditions, particularly when subthreshold presentations are included (13).

Epidemiological studies and psychiatric textbooks consistently report much lower prevalence rates for bipolar disorders compared to depression (14). In the introductory section on bipolar affective disorder in the Synopsis of Psychiatry (15), the authors describe a conceptual debate. One school views affective disorder as a continuum ranging from unipolar depression at one end, through bipolar disorder in the middle, and mania at the other end. Another proposes a bipolar spectrum encompassing Bipolar I, Bipolar II, and recurrent depression. Bipolar I Disorder appears in approximately 1% of children and adolescents, and up to 10% of patients with an initial diagnosis of major depressive disorder (MDD) will develop a manic episode within 10 years of their first depressive episode. The average age of first manic episode is reported to be 32, with most patients experiencing numerous depressive episodes beforehand. As many as 80% of women and 70% of men with Bipolar I Disorder begin with recurrent depressive episodes, while only 10–20% develop manic episodes exclusively (14, 15).

Studies have reported a trend of increasing diagnoses of various forms of bipolar disorder. Epidemiological data on bipolar spectrum disorders (BSDs) remain sparse. Diagnosing hypomanic states is very difficult-even in clinical settings-and virtually impossible in non-clinical interviews or self-report questionnaires. Hypomanic symptoms often go unrecognized by patients, who may not perceive these states as pathological. In fact, hypomania is frequently associated with enhanced productivity, elevated mood, increased energy, and positive social reinforcement-including a heightened ability to achieve personal goals. Further complicating the diagnostic process are mood fluctuations with accompanying irritability, a core feature of hypomania.

These findings indicate that point-prevalence studies may systematically underestimate lifetime bipolarity, particularly in early or subthreshold presentations.

Further complicating the diagnostic process are mood fluctuations accompanied by marked irritability, which represents a frequent and clinically significant manifestation of hypomania and manic states. Contemporary diagnostic frameworks and empirical studies indicate that irritability may be as prominent as euphoric mood in bipolar spectrum disorders, particularly in mixed, dysphoric, or agitated presentations, and is increasingly recognized as a core affective polarity within bipolar psychopathology rather than a nonspecific symptom (16–18).

These features hinder differential diagnosis-especially when distinguishing hypomania from personality disorder symptoms, particularly those belonging to Cluster B (histrionic, borderline, and narcissistic personality disorders) (19).

Studies show that patients with comorbid personality disorders experience affective fluctuations at an earlier age. They also display higher rates of suicidal behavior compared to patients with “pure” BSDs. Some studies report comorbidity rates as high as 32.5% between Bipolar II Disorder and personality disorders (20).

Elevated rates of bipolar spectrum features have been reported among patients diagnosed with atypical or treatment-resistant depression. Early clinical studies, including small inpatient and day-hospital samples, suggested particularly high proportions of bipolarity within these populations; however, such findings were derived from limited cohorts and should be interpreted in their historical and methodological context. More recent investigations using broader samples and contemporary diagnostic frameworks indicate substantial variability in prevalence estimates, with bipolar spectrum features identified in a significant – but not uniform – subset of patients with atypical or treatment-resistant depressive presentations. Collectively, these data suggest an increased likelihood of bipolar spectrum pathology in these clinical groups, while underscoring the need for cautious interpretation and longitudinal diagnostic confirmation rather than reliance on fixed prevalence figures (17, 21–23).

High rates of Bipolar II Disorder have also been reported in individuals with obsessive-compulsive disorder, panic disorder, social anxiety disorder, body dysmorphic disorder, and substance use disorders (24).

## 4 Diagnosis of BPSD subgroups

Clinical operationalization of a bipolar spectrum model requires emphasis on longitudinal course, episodicity, family history, and treatment response patterns; dimensional assessments may enhance diagnostic reliability and inter-rater agreement.

When diagnosing bipolar affective disorder, several clinical findings may increase the likelihood of the condition. A key factor is age of onset. The earlier mood changes emerge – particularly during adolescence – the higher the probability of bipolar affective disorder. For example, in a cohort of children aged 6 to 12 (mean age = 10) diagnosed with major depressive disorder, by age 20 (after a 10-year follow-up) 48.6% were diagnosed with bipolar disorder (25).

Offspring of bipolar parents are at elevated risk of developing BPSD (26). Positive family history of bipolar affective disorder and postpartum depression confers significant predictive value. Numerous studies have demonstrated a strong association between early-onset bipolar disorder and familial history (27). Genetically, the offspring of individuals with bipolar disorder are more likely to develop a major psychiatric disorder (28).

Though challenging, past or present manic or hypomanic episodes should be assessed. In about 80% of patients with bipolar disorder, the illness begins with depressive episodes, and the first manic episode may not appear until roughly 10 years later. For example, in a Dutch high-risk cohort of people with a family history of bipolar disorder, 15 of 17 participants who later developed bipolar disorder started with depression or dysthymia. In some cases, only recurrent depression occurs during this interval (29, 30).

Postpartum depression is more common in patients with bipolar disorder. In Conejo-Galindo et al.’s study, more than 50% of women diagnosed with postpartum depression actually suffered from BD (30), yet only 10% had a previous diagnosis (31). While around 15% of women experience postpartum depression, most are still diagnosed with unipolar depression despite evidence indicating that at least half, and likely more, belong to the bipolar spectrum (32).

Accurate diagnosis of postpartum is essential, as treatment for bipolar depression differs significantly from that of unipolar depression. Clinicians should consider BD in postpartum women presenting with atypical depressive symptoms, family history of BD, or poor response to antidepressants. Antidepressants may not only be ineffective but may worsen the patient’s psychiatric state. A 2020 meta-analysis found that women whose first bipolar episode occurred postpartum (12.2%) had more stable family lives, more children, higher likelihood of BD-II, lower rates of suicide attempts and fewer depressive episodes (33). These findings underscore the importance of screening for BD in postpartum women – an assessment rarely conducted by psychiatrists or gynecologists.

A further clinical feature that may raise suspicion for bipolar spectrum pathology is a relatively abrupt change in affective state, particularly when a depressive episode emerges suddenly and in the absence of an identifiable precipitating factor. Several longitudinal and comparative studies suggest that such unprovoked and rapid onset – especially when accompanied by psychomotor agitation, mood lability, or early functional impairment – occurs more frequently in bipolar disorder than in unipolar major depressive disorder.

In contrast, abrupt onset in unipolar depression is more commonly observed in the context of psychosocial stressors, reactive episodes, or clearly identifiable life events. Accordingly, a sudden depressive shift without an evident external trigger should be regarded as a probabilistic clinical clue suggestive of bipolar depression rather than as a specific diagnostic marker. Its interpretive value increases when considered alongside longitudinal course, family history, prior mood elevation, and treatment response (34–36).

In everyday clinical practice, treating physicians frequently encounter abrupt affective shifts in patients with bipolar spectrum disorders, wherein transitions to depression or mania may occur within hours rather than over days or weeks. Such rapid changes in mood state are often reported by patients as sudden and internally driven, without a clearly identifiable precipitating psychosocial factor.

Patients often relate to metaphors such as “a light switch turning on or off”. Abrupt shifts suggest a pathophysiology fundamentally distinct from unipolar depression (as per the Down-Regulation Theory). Therefore, antidepressants are often ineffective in bipolar depression. This sudden symptom onset may indicate involvement of ionotropic neurotransmitter receptors (e.g., ligand-gated ion channels). It is not surprising that the sudden symptom onset as “twin brother” is one of the key symptoms in a group of patients with Borderline Personality Disorder (BPD) and in patients with ADHD.

The occurrence of hypomanic or manic symptoms during antidepressant treatment represents a clinically important but heterogeneous phenomenon. In some patients, antidepressant-induced hypomania may serve as an indicator of underlying bipolar spectrum vulnerability, particularly when symptoms persist beyond drug discontinuation, recur across treatments, or are accompanied by a characteristic longitudinal course and family history of bipolar disorder.

However, not all mood elevations emerging during antidepressant exposure reflect true hypomanic switching. Transient activation effects – such as increased anxiety, insomnia, irritability, or psychomotor agitation – may occur in both unipolar and bipolar depression and do not necessarily imply bipolarity. In addition, antidepressants may contribute to mood destabilization in susceptible individuals, leading to mixed or dysphoric states without fulfilling full criteria for hypomania or mania (37–39).

Accordingly, antidepressant-associated mood elevation should not be considered a diagnostic marker in isolation. Its clinical significance depends on phenomenological characteristics, temporal relationship to treatment, persistence after medication changes, and the broader longitudinal and familial context. Contemporary guidelines therefore emphasize cautious interpretation of antidepressant-induced hypomania, integrating it into a comprehensive diagnostic assessment rather than treating it as a pathognomonic sign of bipolar disorder.

Epidemiological data show that over 30% of MDD patients fail to respond to traditional antidepressants (40). According to the STAR*D study, 10–20% of MDD patients remained symptomatic for over two years, despite multiple treatment trials (41). While antidepressants may benefit some MDD patients, only one-third achieve full remission (42).

In contemporary treatment paradigms, remission rather than partial response has become the accepted therapeutic goal in major depressive disorder. However, remission rates remain substantially lower than response rates, and a considerable proportion of patients follow recurrent or persistent depressive trajectories despite adequate pharmacological trials. While such patterns do not in themselves establish bipolarity, they underscore the possibility that strictly unipolar formulations may insufficiently capture longitudinal affective heterogeneity in a subset of cases.

Understanding the natural course of illness is crucial. Untreated depressive episodes can last from two months to several years (average ~5–6 months). One-third of patients recover within a year (43). About 50% of bipolar depressive episodes last between 2–7 months (median=3 months) (34). Thus, many of these patients may in fact suffer from undiagnosed bipolar depression, with spontaneous remission wrongly attributed to treatment. Likewise, apparent treatment resistance may stem from inappropriate interventions following incorrect diagnosis.

The duration of untreated depressive episodes shows marked variability across individuals and diagnostic groups. Contemporary longitudinal and population-based studies indicate that many untreated major depressive episodes remit within several months, with a substantial proportion of patients achieving recovery within the first year. Nevertheless, a significant minority experience prolonged episodes extending beyond twelve months, particularly in the presence of recurrent illness, comorbidity, or bipolar spectrum features. In bipolar disorder, depressive episodes tend to be more recurrent and contribute disproportionately to cumulative illness burden over time, underscoring the clinical importance of early recognition and appropriate treatment (44–47).

Hypermnesia (selective superior memory) is common among BPSD patients. These individuals often report unusually good recall for trivial details which may support academic or professional success. Those with BD-II frequently achieve high levels of functioning due to prolonged hypomanic phases.

Impulsivity is another defining trait of BD and persists even during euthymic intervals (48); this is a common phenomenon that overlaps with bipolar affective disorder, attention deficit hyperactivity disorder, and BPD.

Cyclic depressive episodes, especially under proper treatment and monitoring, strongly suggest bipolar rather than unipolar depression. Many bipolar patients are misdiagnosed with recurrent MDD and are treated with antidepressants that yield limited or no benefit (49). A naturalistic study showed that 37% of BD patients were initially misdiagnosed with unipolar depression (50).

The present analysis suggests that careful longitudinal assessment and consideration of bipolar spectrum features are essential in patients presenting with recurrent or atypical depressive episodes, particularly in the context of treatment resistance or mood instability.

In the 1970s, Kupfer noted that one subtype of MDD featured family history and personality traits consistent with BD, good lithium response, and poor antidepressant response (51). Loss of antidepressant efficacy or inconsistent response over time may indicate bipolar depression. These patients often respond well to mood stabilizers, even without antidepressants (52). (**Table 2**).

**Table 2:** Possible markers of bipolar disorder.

1. Recurrent depression 2. Treatment resistant depression 3. Abrupt onset 4. Impulsivity 5. Hypermnesia 6. Family history bipolar 7. Patients or family history peripartum depression

## 5 Comorbidity in BPSD

In the Epidemiologic Catchment Area (ECA) study, the lifetime prevalence of substance-use disorders, panic disorder, and OCD was approximately twice as high in patients with bipolar disorder (61%, 21%, and 21%, respectively) compared to those with unipolar depression (27%, 10%, and 12%, respectively). Among the patients with OCD, 15% suffered from bipolar disorder, and over half were diagnosed with cyclothymia (53).

Substance use disorder, the most prominent comorbid condition in bipolar disorder, is strongly associated with earlier onset of the illness (54). Compared to individuals with bipolar disorder alone, those with comorbid substance use tend to have an earlier onset of illness, more frequent episodes, higher risk of suicidal behavior, and a stronger family history of bipolar disorder. In these cases, the use of drugs, and especially cannabis, is more accurately considered part of the self-treatment rather than a separate drug addiction. Nearly one-third of all cases (35%) develop a substance use disorder in early adolescence (55). In the Course and Outcome of Bipolar Youth (COBY) study 32% were found to develop substance use (56). Yen et al. reported that 12.2% of adolescents suffering from both substance use and bipolar affective disorder met criteria for BPD (57).

Panic disorder was reported in 20.8% of individuals with bipolar disorder, compared to 10% in those with unipolar depression. These patients also tend to experience earlier onset of bipolar disorder (58).

One of the most prevalent comorbid diagnoses in bipolar disorder is attention-deficit/hyperactivity disorder (ADHD). In the COBY study, 58.6% of the sample met diagnostic criteria for ADHD at study entry (59). It is generally difficult to differentiate between early adolescent manifestations of bipolar affective disorder and those of ADHD. Later in life, differential diagnosis between these two disorders remains challenging-especially in cases of bipolar disorder type II. ADHD or anxiety has been shown to be associated with more severe mood symptoms, greater functional impairment and a poorer clinical course (60).

At this stage, we do not consider it critical to define the phenomenon as comorbidity or as a single disorder with subtypes. When affective symptoms dominate, the overlap is better understood as an affective disorder with subgroups. The literature distinguishes between true comorbidity (independent co-occurring conditions) and artificial comorbidity, where overlapping symptoms lead to misdiagnosis, overdiagnosis, or diagnostic inflation. Differentiating true comorbidity from artificial comorbidity requires careful differential diagnosis, adherence to hierarchical guidelines, and awareness that some diagnostic categories may be overly narrow or overlapping (61, 62). From all the above, clearly there is a need for basic, expansive, longer-term prospective research on these subgroups.

## 6 Borderline personality disorder – a diagnostic challenge

While evidence supporting dimensional and hybrid personality models is acknowledged, the present critique targets categorical reification of BPD rather than denial of the clinical phenomena it describes.

The term *Borderline Personality Disorder* was first introduced by the American psychoanalyst Adolf Stern in 1938 to describe patients who did not conform to the prevailing categories of neurosis or psychosis, but appeared to occupy an intermediate clinical position marked by emotional dysregulation, relational instability, and disturbances of self-identity (63).

The concept was further elaborated through the work of Otto Kernberg in the 1960s and 1970s. John Gunderson subsequently played a pivotal role in operationalizing BPD as a distinct diagnostic category during the 1970s and 1980s, contributing to its inclusion in DSM-III (1980). Notably, the DSM working group initially concluded that empirical support for a separate BPD diagnosis was insufficient; the final inclusion followed sustained advocacy efforts, resulting in its classification under *Emotionally Unstable Personality Disorder* (F60.3), with impulsive and borderline subtypes (64).

Clinically, BPD is defined by features such as interpersonal instability, affective lability, intense anger, self-harm, chronic emptiness, and transient dissociation-symptoms that substantially overlap with other psychiatric conditions, particularly bipolar affective disorder. Importantly, BPD criteria largely describe fluctuating behavioral and affective states rather than enduring personality traits, with symptoms often changing over time or remitting entirely (65). Factor-analytic and bifactor studies further demonstrate that BPD criteria lack diagnostic specificity, loading predominantly on a general psychopathology factor rather than on a disorder-specific dimension (66, 67).

Significant diagnostic overlap has also been documented between BPD and autism spectrum disorder, particularly among women, where misdiagnosis has been associated with stigmatization, diagnostic overshadowing, and adverse clinical outcomes (68).

Qualitative phenomenological studies have provided descriptive insights into the subjective experience and meaning-making processes of individuals diagnosed with borderline personality disorder. Such studies, including small-sample, interview-based investigations, are valuable for generating hypotheses and illustrating experiential patterns but are inherently limited in their capacity to establish generalizable or confirmatory evidence. Accordingly, these findings should be interpreted as illustrative and exploratory, complementing rather than substituting larger-scale empirical, epidemiological, or longitudinal research.

Patients labeled with BPD are frequently perceived as “difficult to treat” or “high-risk,” a perception that may contribute to under-recognition of comorbid conditions and misattribution of affective symptoms. Consequently, the diagnostic label itself may interfere with accurate case formulation, treatment planning, and clinical research (**Table 3**).

**Table 3:** Hypothesized bipolar spectrum phenotypes (heuristic, longitudinal model).

Phenotypic cluster	Core clinical features	Required validators	Differential considerations
Bipolar I	Mania + depression	DSM-5 criteria	Psychotic disorders
Bipolar II	Hypomania + depression	DSM-5 criteria	Recurrent MDD
Bipolar with Prominent Panic	Episodic mood disorder + panic episodes	Polarity shifts; family history	Primary panic disorder
Bipolar with Rapid Cycling	≥4 polarity shifts/year	Documented episodicity	Borderline traits; substance use
Ultra-Fast Bipolar Spectrum Phenotype	Rapid affective shifts (hours–days), irritability, impulsivity, relational intensity, self-harm	Longitudinal polarity shifts; family loading; antidepressant sensitivity; mood stabilizer response	BPD; ADHD; trauma-related dysregulation
Bipolar with ADHD Overlap	Episodic mood disorder + attentional dysregulation	Mood symptoms independent of stimulant exposure	Primary ADHD
Bipolar with Antidepressant-Associated Activation	Hypomanic features emerging during treatment and persisting beyond discontinuation	Recurrence pattern; family history	Transient medication side-effects
Bipolar Depression with Persistent Treatment Resistance	Recurrent depression + poor antidepressant response + bipolar validators	Longitudinal instability	Chronic unipolar depression

Heuristic framework; requires prospective validation; not a DSM reclassification.

Treatment considerations within these phenotypic clusters should remain individualized and grounded in longitudinal clinical assessment rather than determined solely by phenotype designation.

Each of these proposed phenotypic clusters may demonstrate differential patterns of treatment response. The following considerations reflect commonly observed clinical tendencies rather than prescriptive first-line treatment mandates, and should always be individualized based on longitudinal course, functional severity, comorbidity profile, and patient-specific factors.

1. Type I phenotype: Frequently responsive to lithium, sometimes in combination with lamotrigine depending on depressive burden and course characteristics.

2. Type II phenotype: Lamotrigine is often considered in light of its antidepressant and mood-stabilizing properties.

3. Panic/Rapid Cycling presentations: Valproic acid may be beneficial in patients demonstrating cycling vulnerability or anxiety-linked affective shifts, including panic attaks.

4. Ultra-Fast phenotype (longitudinal affective presentation overlapping with selected cases currently diagnosed as BPD): Treatment strategies may involve combination mood-stabilizing approaches tailored to the dominant polarity and longitudinal pattern, rather than fixed pharmacological algorithms.

5. ADHD comorbidity: Psychostimulants may be considered cautiously when attentional impairment persists following affective stabilization.

6. Predominantly manic presentations: Lithium remains a well-established option in classic manic trajectories.

7. Antidepressant-associated polarity shifts: Re-evaluation of antidepressant exposure is recommended; mood stabilization strategies may be prioritized depending on longitudinal response patterns.

8. Recurrent treatment-resistant depressive presentations: Combined strategies, including electroconvulsive therapy (ECT) where clinically indicated, may be appropriate in selected severe cases.

This diagnostic ambiguity has yielded inconsistent conclusions in the literature. Ruggero et al. (69) reported that approximately 40% of individuals diagnosed with BPD had previously received an alternative psychiatric diagnosis.

They concluded that underdiagnosis of bipolar spectrum disorders may be driven, at least in part, by overdiagnosis of borderline personality disorder, whereby affective instability and interpersonal dysregulation are preferentially conceptualized as personality pathology rather than as manifestations of bipolar spectrum illness.

Genetic and family studies further underscore this overlap (13). Family studies have demonstrated elevated rates of bipolar disorders, rather than affective disorders broadly, among first-degree relatives of patients diagnosed with borderline personality disorder. Approximately 10% of first-degree relatives meet criteria for bipolar disorder, representing a markedly increased risk compared with the general population (70).

The longitudinal course of BPD also diverges from that of most personality disorders. Whereas personality traits typically consolidate with age, many individuals diagnosed with BPD demonstrate symptomatic improvement over time. As noted in *Kaplan & Sadock’s Synopsis of Psychiatry*, “in the fourth and fifth decades, these individuals tend to attain greater stability in their relationships and functioning” (19, p. 2156), challenging the assumption that BPD reflects a fixed and lifelong personality style.

Another major area of debate concerns treatment practices. Although psychotherapy is widely regarded as the treatment of choice for BPD, combined psychotherapeutic and pharmacological approaches are frequently employed. While modalities such as Dialectical Behavior Therapy can reduce impulsivity and emotional dysregulation, a substantial proportion of patients do not respond adequately to psychotherapy alone (71, 72). Epidemiological data indicate that the majority of individuals diagnosed with BPD receive psychotropic medication, often in polypharmacy regimens (73).

The observed discrepancy between guideline recommendations and real-world pharmacological practice should not be interpreted exclusively as evidence of diagnostic misclassification or conceptual inconsistency. Alternative explanations merit consideration, including off-label prescribing for severe affective instability, impulsivity, aggression, insomnia, or comorbid anxiety, as well as limited access to specialized psychotherapeutic care and systemic constraints within mental-health services.

In recent critical commentaries, Tyrer and Mulder (74, 75) have questioned the conceptual validity of BPD as a personality disorder, arguing that its defining features reflect fluctuating affective and behavioral states rather than enduring traits, and advocating for dimensional models of personality pathology as adopted in ICD-11.

While a substantial body of literature proposes that a subset of patients diagnosed with borderline personality disorder may be better conceptualized within a broader bipolar spectrum framework, this position is not universally accepted. Dimensional and hybrid models emphasize interpersonal dysfunction and stress-reactive affective instability, whereas spectrum-based approaches underscore longitudinal mood dysregulation and diagnostic fluidity. A balanced consideration of these perspectives supports a nuanced, non-polemical understanding of affective dysregulation and informs ongoing debates regarding psychiatric classification.

While a substantial body of literature proposes that a subset of patients diagnosed with borderline personality disorder may be better conceptualized within a broader bipolar spectrum framework, this position is not universally accepted. Dimensional and hybrid models emphasize interpersonal dysfunction and stress-reactive affective instability, whereas spectrum-based approaches underscore longitudinal mood dysregulation and diagnostic fluidity.

A balanced consideration of these perspectives supports a nuanced, non-polemical understanding of affective instability.

Importantly, the present manuscript does not advocate wholesale reclassification of borderline personality disorder. Rather, it suggests that a clinically meaningful subset of individuals currently diagnosed with BPD may represent a longitudinal affective phenotype overlapping with bipolar spectrum presentations, particularly when polarity shifts, family loading, episodicity, and treatment-response validators are documented.

## 7 The theory

The true prevalence of bipolar spectrum disorder (BPSD) is vastly underestimated. Many patients currently diagnosed with other psychiatric conditions-particularly (BPD) and treatment-resistant depression-may fall within the bipolar spectrum.

Accumulating epidemiological, longitudinal, and clinical evidence suggests that prevalence estimates of bipolar spectrum disorders based on narrowly defined categorical criteria may substantially underestimate the true lifetime burden of bipolarity. Population-based surveys incorporating subthreshold presentations, longitudinal diagnostic transitions, and mixed affective states consistently report higher rates of bipolar spectrum features than traditional DSM-based estimates. Taken together, these findings support the hypothesis that bipolar spectrum disorders are under-recognized in routine epidemiological assessments, particularly when early, attenuated, or diagnostically unstable presentations are excluded (13, 21, 48).

### 7.1 Evaluation of the theory

The diagnostic evolution of bipolar disorder, from ancient notions of melancholia and mania to Kraepelin’s manic-depressive insanity and the more recent DSM-defined bipolar subtypes, reflects ongoing shifts in conceptualization rather than definitive biological demarcation. This conceptual fluidity has persisted into modern nosology, where the distinction between bipolar I and II disorders, and emerging recognition of subthreshold or atypical presentations, underscore the heterogeneity of bipolar pathology.

Epidemiological estimates of a 1–2% lifetime prevalence for bipolar disorder appear insufficient. Studies have highlighted high rates of misdiagnosis, particularly in cases of recurrent depression, treatment resistance, postpartum depression, or impulsivity-features often dismissed as secondary rather than pathognomonic. Data showing that up to 48.6% of children initially diagnosed with MDD subsequently met criteria for bipolar disorder suggest that longitudinal trajectories are crucial for accurate nosology (25). These observations raise the methodological question of how prevalence estimates might change if longitudinal spectrum phenomena, rather than strictly categorical thresholds, were systematically incorporated into epidemiological modeling.

The high co-occurrence of bipolar disorder with anxiety disorders, ADHD, OCD, substance use disorders and personality disorders-especially borderline-raises important questions regarding overlapping pathophysiology, shared genetic vulnerabilities, and diagnostic boundaries.

To clarify the rationale underlying higher spectrum-based prevalence hypotheses and building upon epidemiological reconceptualizations proposed by Angst and colleagues (21) as well as large-scale survey data from Merikangas et al. (13), a conceptual decomposition model may be considered.

Established epidemiological data support approximately 1–2% prevalence for Bipolar I disorder and approximately 3–5% for Bipolar II disorder (13, 21).

Additional spectrum contributors may theoretically include:

• X% individuals with recurrent subthreshold hypomanic states that fail to meet full DSM duration or impairment thresholds, consistent with proposals for minor bipolar or subthreshold bipolarity (21).

• Y% individuals demonstrating antidepressant-associated polarity shifts that persist or recur beyond medication exposure, as discussed in longitudinal and treatment-response studies (22, 37).

• Z% individuals with recurrent depressive episodes accompanied by bipolar validators (e.g., strong family loading, episodicity, mixed features, mood stabilizer responsiveness), findings reflected in population-based surveys and prospective cohort data (13, 25).

Under restrictive categorical thresholds, these intermediate groups are typically redistributed into unipolar depression, anxiety disorders, ADHD, or personality pathology. When conceptualized longitudinally, however, they may represent attenuated or incomplete bipolar spectrum presentations.

This decomposition is presented as a heuristic theoretical model rather than as a definitive epidemiological calculation. Its purpose is to illustrate how cumulative spectrum phenomena may plausibly yield higher prevalence estimates, pending formal longitudinal population validation.

Importantly, this model does not imply diagnostic inflation nor categorical expansion of bipolar disorder. Rather, it highlights the possibility that restrictive cross-sectional thresholds may under-recognize longitudinal affective phenotypes. Any adjustment of prevalence estimates would require prospective validation in large-scale, population-based cohorts employing standardized operational criteria.

## 8 Discussion

The many contemporary studies, some of which are discussed in this article argue that BPD may be an artifact of outdated nosological frameworks. The lack of trait stability, the overlap with BPSD symptomatology, and poor construct validity in factor-analytic research all point to BPD as a state-based syndrome better conceptualized within the bipolar spectrum rather than as a discrete personality disorder. This reinterpretation aligns with contemporary movements in the ICD-11 and the NIMH’s Research Domain Criteria (RDoC), which emphasize dimensional over categorical diagnoses.

Furthermore, the proposed subtype taxonomy of bipolar affective disorder reflects both clinical realities and treatment-responsive patterns. Subtypes such as “bipolar with panic attacks”, “ADHD comorbidity”, “ultra-fast cycling”, and “antidepressant-induced hypomania or mania” were derived from decades of clinical observation and treatment response profiles. Recognizing these phenotypes may facilitate more precise treatment strategies, reduce polypharmacy, and prevent the iatrogenic consequences of misdiagnosis.

Treatment considerations within these proposed phenotypic clusters should remain individualized and grounded in longitudinal clinical assessment, functional impact, and documented treatment-response patterns, rather than determined solely by phenotype designation.

Below is a description of the clinical picture of the proposed subgroups of the bipolar spectrum.

1. Bipolar affective disorder with panic attacks – the main symptoms of bipolar affective disorder and, alongside them, symptoms and signs of panic attacks, both past and present, often beginning in adolescence, sometimes appearing at night – with all the symptoms of panic.

2. Bipolar with ADHD – the main symptoms of bipolar affective disorder and, alongside them, symptoms and signs of ADHD, beginning in adolescence.

Revised Version (Reviewer-Adjusted)

3. Bipolar Mood Disorder, Ultra-Fast Type (Longitudinal affective phenotype overlapping with selected presentations currently diagnosed as BPD)

This proposed phenotype refers to individuals demonstrating core bipolar affective features expressed through very high-frequency mood shifts, sometimes occurring multiple times within a single day. These fluctuations are characterized primarily by marked irritability, impulsivity, affective intensity, and instability in interpersonal functioning. Self-injurious behaviors and chronic feelings of emptiness may also be present.

Importantly, these features alone do not justify reclassification. The proposed formulation applies only when longitudinal evidence of polarity shifts, episodicity, family loading for bipolar disorder, antidepressant sensitivity, or mood stabilizer responsiveness supports a bipolar-spectrum interpretation. In the absence of such validators, stress-reactive or trauma-related affective instability should not be subsumed under bipolar spectrum conceptualization.

Operationally, spectrum attribution within this phenotype requires longitudinal evidence of episodicity, polarity shifts, family loading for bipolar disorder, or reproducible treatment-response patterns. High-frequency affective reactivity in the absence of such validators-particularly when clearly stress-triggered or trauma-related-should not be interpreted as bipolar spectrum pathology.

4. Bipolar Mood Disorder with Treatment-Resistant Depressive Episodes – for at least 5 and up to 10 years, repeated depressive episodes with severe depression (melancholia), often after repeated changes of antidepressants taken in adequate doses and adequate time frames; there may be a positive “reaction” to one of them but subsequently, despite taking the drug, depression develops again and no longer responds to this drug or to an increased dose. The medical history includes a family history of psychiatric disorders, mainly affective ones, ADHD, drug addiction, on the female side there may be indications of peripartum depression.

All other subgroups of bipolar disorder proposed in the article are described in the contemporary literature.

The proposed bipolar spectrum subgroups are not intended to replace existing comorbidity-based diagnostic frameworks, nor to reclassify established categorical diagnoses. Rather, their purpose is to address clinically and methodologically relevant dimensions that are insufficiently captured by current diagnostic systems organized primarily around cross-sectional symptom counts and comorbidity labels.

Specifically, the rationale for delineating bipolar spectrum subgroups lies in their capacity to reflect differences in longitudinal course, phenomenological clustering, and patterns of treatment response that frequently cut across traditional diagnostic boundaries. Patients sharing similar comorbidity profiles may nonetheless differ substantially in illness trajectory, affective reactivity, episodicity, and response to pharmacological or psychotherapeutic interventions-differences that are clinically meaningful but often obscured within existing categorical classifications.

In this context, subgrouping is proposed as a heuristic framework aimed at improving clinical formulation rather than as a rigid nosological subdivision. By organizing patients according to recurring patterns of affective instability, episodicity, and course over time, such an approach may enhance diagnostic sensitivity and guide more individualized treatment strategies without negating the relevance of established diagnoses.

Importantly, beyond its immediate clinical utility, the proposed subgrouping may also facilitate future research. Recognition of distinct clinical phenotypes within the bipolar spectrum could allow for more homogeneous sampling in longitudinal, genetic, and neurobiological studies, thereby improving signal detection and reducing heterogeneity-related noise. From this perspective, subgroup differentiation reflects not only observable differences in clinical presentation but also a potential pathway toward more precise investigation of underlying mechanisms as empirical methods and biomarkers continue to evolve.

Operationalization of a bipolar spectrum framework requires emphasis on longitudinal course, episodicity, family history, and treatment response patterns. Structured interviews should be complemented by dimensional assessments to enhance diagnostic reliability and inter-rater agreement. At present, this model should inform clinical reasoning rather than replace existing diagnostic systems.

### 8.1 Risks of overdiagnosis

Expansion of the bipolar spectrum entails risks of overdiagnosis and pharmacological overtreatment, necessitating conservative diagnostic thresholds and longitudinal confirmation.

Expansion of the bipolar spectrum carries inherent risks, including pathologization of normative affective variability and pharmacological overtreatment. Mood stabilizers and antipsychotics carry significant side-effect burdens, underscoring the ethical necessity of conservative thresholds and longitudinal confirmation before diagnosis.

Diagnostic expansion without methodological rigor may increase healthcare utilization and strain mental health resources. Spectrum-based approaches must therefore be balanced by clear safeguards against diagnostic inflation.

## 9 Conclusions

The suggestion that bipolar spectrum prevalence may exceed traditionally reported rates is grounded in converging clinical observations accumulated over decades: prolonged diagnostic delays, misattribution of spontaneous remissions, under-recognition of bipolar presentations in women and adolescents, recurrent depressive episodes repeatedly treated as unipolar illness, and the frequent classification of affective instability under personality-based diagnoses. Collectively, these patterns suggest that prevailing diagnostic systems-primarily structured around cross-sectional symptom thresholds-may insufficiently capture the longitudinal architecture and developmental trajectory of affective illness.

Within this context, the proposed 15–20% prevalence range should be interpreted as a theoretical upper-bound hypothesis rather than as a definitive epidemiological claim. It represents a conceptual modeling exercise derived from cumulative clinical observation, longitudinal follow-up, and extrapolation from subthreshold affective phenomena. Its purpose is not to assert a fixed population parameter, but to illustrate how systematic incorporation of longitudinal course, diagnostic migration, antidepressant-associated polarity shifts, and attenuated spectrum presentations may plausibly alter lifetime prevalence calculations. Any formal revision of epidemiological estimates would necessarily require prospective validation in large-scale, population-based cohorts employing standardized operational criteria capable of capturing diagnostic transitions over time.

Importantly, this analysis does not advocate indiscriminate expansion of bipolar diagnosis nor the categorical relabeling of all affective instability. Rather, it underscores the methodological tension between static cross-sectional classification and longitudinal phenomenology. Careful evaluation of episodicity, polarity shifts, family loading, treatment-response patterns, and developmental course remains essential. Conceptual refinement must proceed alongside methodological restraint and ethical vigilance to avoid both under-recognition and diagnostic inflation.

The dimensional reconfiguration of personality pathology in ICD-11 reflects a broader movement within psychiatry toward course-sensitive and severity-based formulations. In this evolving nosological landscape, the present review does not deny the reality of personality-related suffering nor propose categorical elimination of the borderline personality construct. Instead, it raises the possibility that rigid diagnostic boundaries – particularly in the presence of substantial longitudinal affective overlap – may obscure clinically meaningful mechanisms operating across traditionally separated domains. In a subset of patients, a strictly personality-based formulation may fail to account for recurrent polarity shifts and spectrum-consistent trajectories that carry implications for prognosis and treatment strategy.

From a research perspective, greater attention to longitudinal phenotypes may reduce heterogeneity, improve cohort stratification in genetic and neurobiological studies, and enhance the interpretability of treatment-response data. Clinically, prioritizing affective trajectory and illness course over static diagnostic labeling may facilitate earlier recognition of bipolar spectrum conditions, reduce iatrogenic consequences of misclassification, and promote more individualized therapeutic planning.

In conclusion, this review challenges the sufficiency of narrow categorical prevalence estimates and invites reconsideration of whether current diagnostic boundaries adequately reflect the dynamic and longitudinal nature of affective psychopathology. It proposes a cautious, empirically testable refinement of bipolar spectrum conceptualization – one that seeks improved diagnostic coherence, theoretical integration, and patient-centered precision while preserving meaningful clinical distinctions and methodological rigor.

Ultimately, refinement of bipolar spectrum conceptualization should be guided not by categorical rigidity nor by expansive impulse, but by careful longitudinal observation, empirical scrutiny, and sustained commitment to clinical precision.
